# PiPred – a deep-learning method for prediction of π-helices in protein sequences

**DOI:** 10.1038/s41598-019-43189-4

**Published:** 2019-05-03

**Authors:** Jan Ludwiczak, Aleksander Winski, Antonio Marinho da Silva Neto, Krzysztof Szczepaniak, Vikram Alva, Stanislaw Dunin-Horkawicz

**Affiliations:** 1 Laboratory of Structural Bioinformatics, Centre of New Technologies, University of Warsaw, Banacha 2c, 02-097 Warsaw, Poland; 2 Laboratory of Bioinformatics, Nencki Institute of Experimental Biology, Pasteura 3, 02-093 Warsaw, Poland; 3 Department of Protein Evolution, Max-Planck-Institute for Developmental Biology, Max-Planck-Ring 5, 72076 Tübingen, Germany

**Keywords:** Software, Sequence annotation, Protein structure predictions

## Abstract

Canonical π-helices are short, relatively unstable secondary structure elements found in proteins. They comprise seven or more residues and are present in 15% of all known protein structures, often in functionally important regions such as ligand- and ion-binding sites. Given their similarity to α-helices, the prediction of π-helices is a challenging task and none of the currently available secondary structure prediction methods tackle it. Here, we present PiPred, a neural network-based tool for predicting π-helices in protein sequences. By performing a rigorous benchmark we show that PiPred can detect π-helices with a per-residue precision of 48% and sensitivity of 46%. Interestingly, some of the α-helices mispredicted by PiPred as π-helices exhibit a geometry characteristic of π-helices. Also, despite being trained only with canonical π-helices, PiPred can identify 6-residue-long α/π-bulges. These observations suggest an even higher effective precision of the method and demonstrate that π-helices, α/π-bulges, and other helical deformations may impose similar constraints on sequences. PiPred is freely accessible at: https://toolkit.tuebingen.mpg.de/#/tools/quick2d. A standalone version is available for download at: https://github.com/labstructbioinf/PiPred, where we also provide the CB6133, CB513, CASP10, and CASP11 datasets, commonly used for training and validation of secondary structure prediction methods, with correctly annotated π-helices.

## Introduction

**Figure 1:**
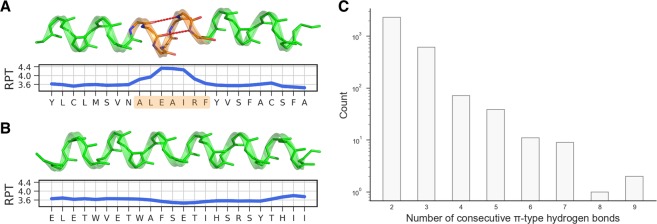
Exemplary backbone structures of (**A**) a π-helix (PDB code: 1MXR, chain A, residues 194–217) and (**B**) an α-helix (PDB code: 1MXR, A, 103–126). Plots below the structures indicate the number of residues per helical turn (RPT). The π-helical region is shown in orange and *i* → *i* + 5 hydrogen bonds are indicated with red dashed lines (*i* → *i* + 4 hydrogen bonds are not shown). (**C**) The length distribution of ~3,000 representative π-helices. Note that the counts are shown in logarithmic scale.

Helices, dominant protein secondary structure elements, are defined by the recurring pattern of the hydrogen bonds between the amide hydrogen (NH) and the carbonyl oxygen (CO) atoms. In α-helices, the most abundant type of helices, this interaction occurs between residues in positions *i* and *i* + 4 in the amino acid sequence. Unlike α-helices, π-helices, a less frequent type of helices, contain hydrogen bonds between residues in positions *i* and *i* + 5 (Fig. 1). Canonical π-helices are characterized by the presence of at least two π-type (*i* → *i* + 5) hydrogen bonds and thus the minimal length of a π-helix is seven residues^1^. Shorter, six-residue-long segments containing a single π-type hydrogen bond are referred to as α/π-bulges and should not be confused with canonical π-helices. The relative instability of π-helices is attributed to unfavorable dihedral angles^2^, weaker van der Waals interactions in the core of the helix^3^, and the large entropic cost of aligning five residues to form π-type hydrogen bonds^3^. As a consequence, most π-helices comprise just seven residues (two consecutive π-type hydrogen bonds); however, examples comprising even 14 residues (nine consecutive π-type hydrogen bonds) have also been identified (Fig. 1).

Evolutionarily, π-helices are thought to have originated through the insertion of single residues into α-helical regions^1^. Despite the fact that such insertions are estimated to have a destabilizing effect, they have been identified in about 15% of all known protein structures, suggesting that they must provide a functional advantage. Indeed, π-helices are frequently found in functionally important regions such as ligand-binding sites^1,4^ as well as transmembrane helical domains such as those of G-protein coupled receptors^5,6^. Given the functional importance of π-helices, it is crucial to develop computational tools for their prediction in structures or based on sequence information. The most popular tools for structure-based annotation of secondary elements, such as DSSP^7^ or STRIDE^8^, tend to favor α-helices over π-helices, frequently resulting in the absence of π-helices in assignments (recently DSSP was corrected to account for this problem^9^). However, several dedicated methods for the annotation of π-helices in protein structures have been developed^1,10,11^, providing the possibility of identifying π-helices that are missed by the general-purpose methods.

In comparison to α-helices, π-helices exhibit different amino acid preferences^12^ – aromatic and large aliphatic amino acids are preferred at the termini, whereas polar amino acids, particularly asparagines, tend to be present in the center^11^. Moreover, proline residues are frequently found directly after π-helices and have been suggested to promote the termination of the π-helical structure. Considering the presence of such sequence hallmarks, the prediction of π-helices directly from sequence appears to be possible.

Modern prediction methods, frequently utilizing neural networks and deep learning approaches, achieve accuracies in the range of 75% to 85% for the 3-state secondary structure prediction problem (H: α-helix, E: β-strand, and C: coil) and up to 70% for the 8-state case that considers five additional secondary structure elements: G: 3_10_-helix, I: π-helix, T: turn, B: β-bridge, and S: bend)^13,14^. However, none of the state-of-the-art 8-state predictors such as DeepCNF^15^, DCRNN^16^, CNNH_PSS^17^, C8-Scorpion^18^, GSN^19^, RaptorXss8^20^, and SSPro8^21^ is capable of predicting π-helices, i. e. the accuracy of these predictors for π-helix class (“I”) is zero. This can be attributed to the properties of the datasets commonly used in the secondary structure prediction problems, like CB6133^19,22^ or CB513^19,23^, which contain only a small number of π-helices due to inaccuracies in DSSP^9^. For example the original CB6133 dataset includes only 42 sequences containing a π-helix, which constitutes 0.7% of all sequences in the dataset, about 20 times lower than the fraction of the π-helices in PDB structures (for more details see Supplementary Table 2). The only method that is capable of predicting π-helices is limited to those occurring in transmembrane proteins^24^.

Here, we describe PiPred, a rigorously validated deep learning-based tool, trained on 20,295 diverse sequences containing 3,032 canonical π-helices with seven or more residues. For a given protein sequence, PiPred predicts the per-residue probability of the occurrence of π-helices and the three other basic secondary structure elements (α-helices, β-strands, and unstructured regions). Moreover, we show that despite being trained with canonical π-helices, PiPred can also predict some six-residue-long π/α-bulges and other helical distortions. PiPred is part of the Quick2D tool offered by the MPI Bioinformatics Toolkit^25^ and a standalone version can be obtained from https://github.com/labstructbioinf/PiPred.

Additionally, for some commonly used datasets in the development of secondary structure prediction methods (e.g., CB6133, CB513), we offer updated versions with correctly annotated π-helices (Supplementary Table 2; https://lbs.cent.uw.edu.pl/pipred). The use of these updated datasets in the development of new secondary structure prediction methods should result in improved detection of π-helices. In fact, as a proof of concept, using the corrected datasets we retrained two state-of-the-art 8-state secondary structure prediction methods, CNNH_PSS and DCRNN, to be able to detect π-helices, yet with accuracies considerably worse than that of PiPred.

## Results and Discussion

### Functional importance of π-helices in protein structures

To assess the functional role of π-helices, we surveyed 2,555 representative π-helices present in protein structures co-crystallized with ligands and found that 24% of them interact with at least one ligand, most frequently with protoporphyrin IX and its derivatives (e.g. heme, chlorophyll), nucleoside derivatives (e.g. NAD, NADP, FAD), and various ions (e.g. phosphate, sulfate, zinc, and iron). Moreover, examination of 237 representative transmembrane (TM) structures obtained from PDBTM database^26^ revealed that 45% of them contain at least one π-helix. Curiously, π-helices found in TM domains frequently (42%) also interact with ligands such as retinal and chlorophyll. To systematically investigate the association between the presence of π-helices and biological functions, we performed Gene Ontology (GO) enrichment analysis, with a focus on identifying GO terms overrepresented in proteins containing π-helices. We found that structures containing one or more π-helices are enriched with GO terms such as “oxidation-reduction process” (p-value = 3e-61), “heme binding” (p-value = 7e-18), “nucleotide binding” (p-value = 1e-12), and “metal ion binding” (p-value = 7e-8).

The above results illustrate that π-helices play an important role in binding ligands and in the functioning of helical transmembrane domains. These results are in agreement with previous observations that helical deformations, including π-helices, are frequently present in the proximity of NAD-based cofactors and heme groups^4^, and that π-helices participate in the coordination of ions^10^.

### PiPred predictor

We scanned the structures in the Protein Data Bank for the presence of π-helices and subsequently constructed two sets: a training set comprising 20,295 structures used to train PiPred, a deep learning-based method for predicting π-helices in protein sequences; and an independent test set comprising 2,215 structures (Test set “7”) used to validate its performance. Importantly, to assure fairness of the validation procedure, the test set comprised only sequences that share no more than 30% identity with the sequences of the training set (for detailed statistics on the training and test sets see Supplementary Table 1).

**Figure 2:**
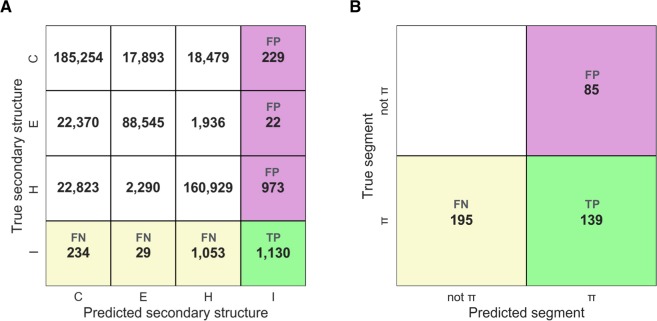
The performance of PiPred in detecting canonical π-helices comprising seven or more residues and containing at least two π-type *i* → *i* + 5 hydrogen bonds. In the confusion matrices, true positives (TP), false negatives (FN), and false positives (FP) are shown in green, yellow, and purple, respectively. (**A**) Performance of the per-residue predictions. Letters indicate secondary structure elements: I: π-helix, H: α-helix, E: β-strand, and C: coil. Numbers indicate residue counts. (**B**) Performance of the per-segment prediction. Numbers indicate segment counts.

**Figure 3:**
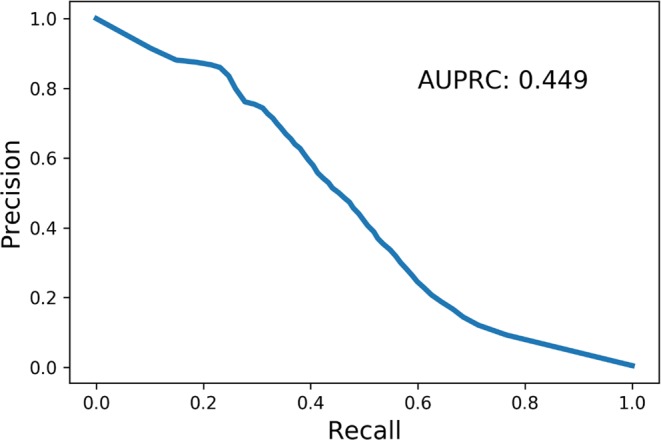
Precision-Recall plot for the detection of π-helices with PiPred.

First, we determined the performance of PiPred in predicting π-helices at single-residue resolution. Every residue of each test set sequence was assigned a label corresponding to the predicted secondary structure type (I – π-helix, H – α-helix, E – β-strand, C – unstructured region). The predicted labels were subsequently compared to the true labels defined based on their three-dimensional structure (for detailed procedure see Methods). True positives were defined as correctly predicted π-helical residues, whereas false negatives and false positives were defined as π-helical residues predicted as non-π-helical and non-π-helical predicted as π-helical, respectively. Using these values, we obtained a precision of 48% and a sensitivity of 46% (Fig. 2A). Moreover, the area under the precision-recall curve (AUPRC) analysis clearly indicated that PiPred is far-better than random assignment and that it is effective at various probability cut-offs (Fig. 3).

In addition to the per-residue performance statistics, in which all residues are treated separately, we used an alternative approach that is based on secondary structure segments. All true π-helical segments present in the test set and predicted by PiPred were pooled together. An overlap by at least one residue between true and predicted π-helices was considered to be a correct prediction (true positive). True π-helices and predicted π-helices that did not overlap with other segments were considered to be false negatives and false positives, respectively. The segment-based approach yielded a precision of 62% and a sensitivity of 42% (Fig. 2B). Most of the incorrect predictions (false negatives and false positives) resulted from the prediction of π-helices as α-helices and *vice versa*: Among 195 π-helical segments missed by PiPred (false negatives), 110 (56%) were mispredicted as α-helices. Similarly, among 85 non-π-helical regions predicted by PiPred as π-helices (false positives), 62 (73%) are α-helices according to structure-based DSSP assignment.

### Common features of π-helices and other helical deformations

**Figure 4:**
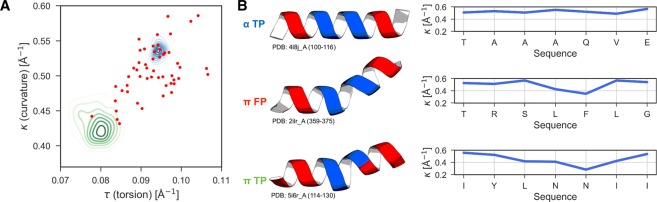
The geometry of helices in the test set. α TP (true positives), π FP (false positives), and π TP indicate α-helices correctly predicted by PiPred as α-helices, α-helices mispredicted as π-helices, and π-helices correctly predicted as π-helices, respectively. (**A**) Curvature and torsion values for α TP and π TP are shown as kernel density estimate plots, and for π FP as points. (**B**) Exemplary structures and corresponding curvature plots. Residue ranges shown in the plots are colored blue in the structures.

To further investigate cases in which PiPred confuses α- and π-helices with each other, we compared the geometry (for details see “Differential geometry analyses” in Methods) and hydrogen bonding patterns of α-helices correctly predicted as α-helices (“α TP”; Fig. 4), α-helices mispredicted as π-helices (“π FP”), and π-helices correctly predicted as π-helices (“π TP”). The geometry of helices was represented as the average per-residue curvature and torsion values: The curvature indicates how much a backbone curve deviates from a straight line at a given point, whereas torsion expresses how much the curve deviates from a plane at a given point. We found that α-helices mispredicted as π-helices (red points in Fig. 4A) frequently show curvature and torsion values lying between those typical for α- and π-helices. In such cases, the curvature and torsion values are lower than those of canonical α-helices, which can be related to the increase of the helical radius and the number of residues per turn, features typical for π-helices and π/α-bulges (Figs 1A and 4B). Despite this resemblance, these structures do not meet the criteria for canonical π-helices, as they lack two (or more) consecutive *i* → *i* + 5 hydrogen bonds with energy lower than alternative *i* → *i* + 4 bonds (see “*Construction of the training and test sets*” in the Methods).

**Figure 5:**
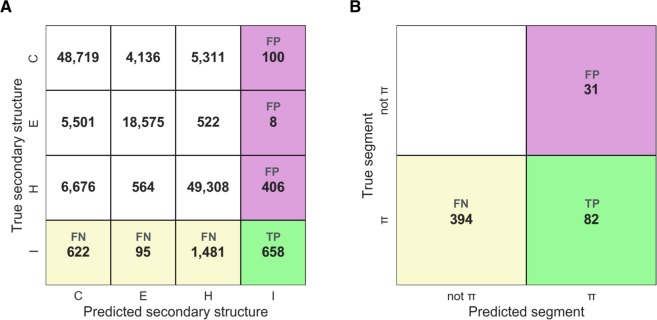
The performance of PiPred in detecting π/α-bulges comprising six residues and containing a single π-type *i* → *i* + 5 hydrogen bond. For details see caption of Fig. 2.

Intrigued by this observation, we decided to perform a systematic analysis of cases in which non-π-helical structures are predicted by PiPred as π-helices. To this end, we constructed an additional test set, Test set “6”, comprising 449 structures with a total of 476 π/α-bulges, i.e. six-residue-long secondary structure motifs characterized by the presence of a single *i* → *i* + 5 hydrogen bond (Supplementary Table 1). Analogously to the benchmark with the standard test set, we assessed the performance of PiPred in per-residue and per-segment detection of π/α-bulges (Fig. 5). The per-residue predictions yielded precision of 56% and sensitivity of 23%, while the per-segment predictions resulted in precision and sensitivity of 73% and 17%, respectively.

The poor sensitivity of PiPred in detecting non-π-helical deformations is to be expected as the method was trained using canonical π-helices comprising seven or more residues. However, the fact that PiPred was exclusively trained using canonical π-helices and yet can detect other helical deformations (Figs 4 and 5) suggests that canonical π-helices, π/α-bulges as well as helical deformations that do not involve π-type *i* → *i* + 5 hydrogen bonding share similar sequence features to some extent. This can be interpreted in the context of the observation that the transition between α and π conformations do occur in protein structures and can be achieved by shifting hydrogen bonding patterns. Such transitions were for example described in transmembrane helices^5,27^ and monooxygenases^1^. In the latter case, they were termed “peristaltic-like shifts” and were associated with binding of a ligand. It is thus possible that some of the false positive predictions indicate that a given region has a propensity for the formation of a canonical π-helix, even though it does not assume such a conformation in an experimental structure.

### Application of the method

To explore the possible applications of PiPred, we used it to scan 7,700 Pfam families with no structural data and comprising at least 30 sequences in the seed alignment. We found that 1200 of them may harbor uncharacterized π-helices, 82 of which are associated with the GO term “integral component of membrane” (enrichment p-value = 5e-12). In addition, a manual inspection of the Pfam descriptions revealed that a further 73 families were membrane-bound proteins, 54 various transporters, and nine G-protein-coupled receptors. Of the remaining families, 342 were annotated as domains of unknown function. A flat-file containing these predictions is available at: https://lbs.cent.uw.edu.pl/pipred.

### Correction of the CB6133, CB5926, CB513, CASP10, and CASP11 datasets

The most routinely used datasets for training and benchmarking secondary structure prediction methods (i.e., *CB6133, CB5926, CB513, CASP10, and CASP11 datasets)* contain only a very limited number of π-helices due to the limitations of DSSP (“Original datasets” in Supplementary Table 2). To address this issue, we corrected the secondary structure assignments in these datasets utilizing an established method for π-helix annotation^1^. Strikingly, these corrections resulted in a nearly 10-fold increase in the number of π-helices (“Updated datasets” in Supplementary Table 2) and did not introduce changes to the distribution of the other secondary structure labels (Supplementary Fig. 1). This prompted us to verify, as a proof of concept, whether two of the current state-of-the-art methods DCRNN and CNNH_PSS, which are not capable of detecting π-helices, will be able to predict them after retraining with the updated datasets. Indeed, after retraining both methods became capable of detecting π-helices, while maintaining the same overall prediction accuracy as their original versions (Table 1). These updated datasets (available at: https://lbs.cent.uw.edu.pl/pipred) should therefore be useful for the development of general-purpose 8-state secondary structure prediction methods that are also capable of detecting π-helices.

**Table 1:** Performance of retrained DCRNN and CNNH_PSS methods on the PiPred “7” test set, and the updated CB513, CASP10 and CASP11 datasets.

	PiPred	DCRNN		CNNH_PSS	
	“π” F1 score^(a)^	Q8 accuracy^(b)^	“π” F1 score	Q8 accuracy	“π” F1 score
Test set ”7”	0.471	0.697	0.314	0.702	0.223
CB513^(c)^	0.538	0.699	0.348	0.701	0.343
CASP10^(c)^	0.557	0.720	0.328	0.725	0.286
CASP11^(c)^	0.559	0.699	0.476	0.705	0.409

^(a)^F1 – harmonic average of the precision and sensitivity; ^(b)^Q8 – index used for the evaluation of secondary structure prediction methods; ^(c)^updated versions of the datasets were used. For details refer to Supplementary Table 2.

To compare the new, retrained versions of DCRNN and CNNH_PSS with PiPred, we tested them on the test set used to assess the performance of PiPred (Test set “7”; Supplementary Table 1) as well as on the updated CB513, CASP10, and CASP11 datasets (Supplementary Table 2). PiPred significantly outperformed both methods in all four tests (Table 1): PiPred “7” test set (p-value = 1e-14 and p-value = 1e-30 for DCRNN and CNNH_PSS, respectively), updated CB513 set (p-value = 0.003 and p-value = 0.0004), updated CASP10 set (p-value = 0.02 and p-value = 0.006), and updated CASP11 set (p-value = 0.06 and p-value = 0.003).

## Conclusions

Our results illustrate that PiPred can be used for the annotation of potential functional sites in proteins since π-helices frequently contribute to protein-ligand interactions. Moreover, the prediction of π-helices and related helical distortions will be helpful for modeling the tertiary structure of transmembrane domains^28^. Finally, we envision that PiPred will also be useful for protein design tasks focused on the creation of ligand-binding pockets and new proteins with ligand-binding potential.

## Methods

### Construction of the training and test sets

The Protein Data Bank structures, grouped into clusters comprising entries that share at least 50% sequence identity, were obtained from https://www.rcsb.org/pages/download/ftp (bc-50.out file). Structures longer than 700 or shorter than 30 residues, with resolution greater than 2.5 Å, and solved by methods other than X-ray crystallography were discarded. This yielded a set comprising 205,527 structures grouped within 24,276 clusters. Secondary structure state was assigned to each residue of each structure using DSSP^7^. Considering the weak performance of DSSP in detecting π-helices, we used a custom π-helix assignment method implemented based on^1^. First, all “I” labels (π-helices) defined by DSSP were substituted with “C” (coils). Next, residue ranges were re-marked as π-helical if (i) at least two π-type hydrogen bonds were present according to DSSP (in the cases where DSSP indicated two alternative hydrogen bonds, e.g. *i* → *i* + 4 and *i* → *i* + 5, the one with lower energy was considered), (ii) at least one of the π-type hydrogen bonds had energy equal or lower than −2.0 kcal/mol, and (iii) torsion angles for all residues in the range fell into the broadly defined helical region (−180° < φ < 0°, −120° < Ψ < 45°). Finally the 8-state assignment was reduced to a 4-state assignment (“H” – helix, containing “G” and “H” labels, “S” – strand, containing “E” and “B” labels, “C” – coil, containing “S”, “T”, and “C” labels, and “I” – π-helix). An analogous approach was used to generate an independent assignment of π/α-bulges, i.e. secondary structure elements comprising just a single *i* → *i* + 5 hydrogen bond. Such six-residue-long regions were defined based on the presence of one π-type hydrogen bond of energy equal or lower than −2.0 kcal/mol and torsion angles fulfilling the criteria listed above.

To build a dataset in which the pairwise sequence identity does not exceed 50%, we selected a representative structure from each of the aforementioned 24,276 clusters. To this end, we used the following procedure: First, from each cluster that does not contain any structure with a π-helix and/or a π/α-bulge, a single representative structure with the lowest resolution was selected. From the remaining clusters, representative structures with the lowest resolution and longest π-helical segment were selected; however, those containing also π/α-bulges were discarded. Such a procedure resulted in an initial dataset comprising 22,510 structures, of which 2,985 contained at least one canonical π-helix and did not contain any π/α-bulge (positive examples set), and 19,525 did not contain π-helices as well as π/α-bulges (negative examples set). From the remaining 1,750 structures, those containing at least one π/α-bulge but no π-helices were selected, filtered to 30% sequence identity, and used to generate a separate “π/α-bulge” set comprising 449 structures.

Sequences corresponding to the 22,959 structures (2,985 positive examples set, 19,525 negative examples set, and 449 “π/α-bulge” set) were used as queries in PSI-BLAST^29^ (E-value < 0.001, three iterations) searches of the NCBI non-redundant protein sequence database filtered to 90% sequence identity for the calculation of position specific scoring matrices (PSSMs). Subsequently, the 22,510 structures of the positive and negative examples sets were merged, shuffled, and randomly assigned to the training and test sets comprising 20,295 and 2,215 sequences, respectively. Importantly, we ensured that all sequences of the test set show no more than 30% similarity to any sequence of the training set. Furthermore, we also ensured that the two datasets contained an equal percentage of the π-helical residues, which amounted to 0.46% of all residues in each set. Detailed statistics for all data sets are shown in Supplementary Table 1.

### Sequence encoding

For encoding sequences and their corresponding PSSM profiles, we used a procedure in which a sequence is encoded as a 700 × 40 matrix, where 700 and 40 corresponds to the maximal sequence length and the number of features associated with every residue, respectively. Out of 40 features, 20 denoted “one-hot” encoded amino acid and another 20 were the PSSM probabilities transformed by the sigmoid function. Finally, if the sequence was shorter than 700 residues it was padded with zeros randomly at the C- or N- terminal ends to match the 700 × 40 matrix size.

### Deep-learning model architecture and training

**Figure 6:**
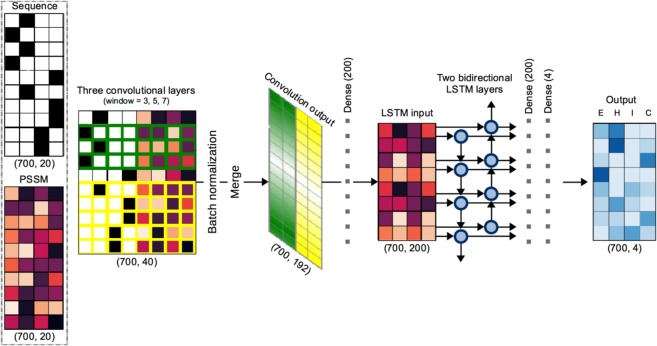
Schematic representation of the prediction model architecture. Numbers below matrices indicate their dimensionality. The input sequence and the corresponding PSSM are encoded as 700 × 40 matrix, which is introduced into three independent convolutional layers with window lengths of 3, 5, and 7 (for clarity only windows of the length of 3 and 5 are shown in green and yellow, respectively). The output of each convolutional layer is normalized and subsequently, all outputs are merged and passed through a dense layer, two LSTM layers, another dense layer, and an output layer. The output is a 700 × 4 matrix where each row denotes probabilities of E (strand), H (α-helix), I (π-helix), and C (unstructured coil) occurring at the given position of the input sequence. For details refer to “Deep-learning model architecture and training” section of Methods.

The cascaded convolutional and recurrent network architecture, based on the DCRNN secondary structure predictor^16^, with minor modifications, was implemented in Keras^30^ using Tensorflow^31^ backend (Fig. 6). Sequences and PSSMs encoded as 700 × 40 matrices (see “Sequence encoding” section above for details) were independently introduced into three 1D convolutional layers (window lengths 3, 5 and 7), each of which contains 64 filters and is activated with the *tanh* function. The output of the three convolutional layers, i.e. three 700 × 64 matrices, were passed through batch normalization layers and concatenated to yield a 700 × 192 matrix. Next, the concatenated matrix was used as an input to a fully-connected layer containing 200 neurons and activated with the ReLU function. To detect the dependencies between distant residues based on the local features extracted by convolutional layers two bidirectional LSTM layers were used. Each consisted 200 neurons and their dropout and recurrent dropout parameters were set to 0.5, and activation and recurrent activation functions were set to *tanh* and sigmoid, respectively. Finally, a dense layer with 200 neurons and the ReLU activation function was used to connect the LSTM with an output layer containing 4 neurons and the softmax activation function. The final output of the network is a vector 700 × 4 indicating the residue-wise probabilities of the 4 secondary structure classes: I – π-helix, H – α-helix, E – β-strand, C – unstructured region. The implementation in Python and Keras is available at: https://github.com/labstructbioinf/PiPred.

The training process involved optimization of the network’s parameters using pairs of encoded sequences and their corresponding correct secondary structure labels. The training process was performed in a 10-fold cross-validation (CV) framework: the training set was randomly divided into 10 equally-sized parts, each containing approximately the same number of π-helical residues. In each CV round, one part served as validation set, whereas the remaining nine together as training set. The training was performed for 50 epochs with the ‘Adam’ optimizer^32^ (the learning rate was set to 0.0003, whereas the remaining parameters were set to their default values) with categorical cross-entropy as the loss function (to account for significant underrepresentation of π-helices, the π-helix class was weighted by a factor of five). From each CV round, the best model (according to the F1-score of π-helix classification) was selected and the resulting 10 models were used to build the final ensemble predictor (PiPred). For each residue of a given sequence and PSSM, PiPred returns four probabilities corresponding to four secondary structure classes (I, H, E, and C). In each position of the input sequence, the predicted secondary structure is defined as the one with the highest probability.

### Function of π-helices

22,510 structures of the initial dataset (positive and negative examples) were used to identify associations between the presence of π-helices and biological functions. The PDB to Gene Ontology (GO) mappings were downloaded from www.geneontology.org/gene-associations/goa_pdb.gaf ^33^ and 11,066 out of 22,510 structures were found to have at least one associated GO term. These structures were analyzed with GOATOOLS^34^ to identify the enrichment of GO terms in 1,773 structures containing one or more π-helices (p-values were adjusted with the Holm method). In addition to the GO enrichment statistics, we analyzed 2,555 π-helices present in structures containing ligands using PLIP^35^.

### Differential geometry analyses

A differential geometry representation of protein backbones was used to evaluate and compare the geometry of (i) π-helices correctly predicted as π-helices, (ii) α-helices correctly predicted as α-helices, and (iii) α-helices mispredicted as π-helices (false positives). To this end, we used the FleXgeo^36^ method, which implements an approach analogous to that used in CHORAL^37^, ARABESQUE^38^, and Polyphony^39^. The protein backbone is represented by a piecewise cubic spline interpolation using the Cα atoms as knots, i.e. as a regular smooth curve $\vec{r}(t)$ parametrized by Cα residue number $t$. According to the Fundamental Theorem of Curves, any regular spatial curve can be fully characterized by its curvature, $\kappa$, and torsion, $\tau$, values as a function of arc length $s$. Given the allowable change of parameters, $s$ and $t$ are related by:

1 $$\frac{ds}{dt}=\Vert \frac{d\vec{r}}{dt}\Vert $$

Therefore, it is possible to calculate $\kappa \,$and $\tau$ using the following equations:

2 $$\kappa =\vec{\kappa }=\Vert \frac{d\vec{T}}{ds}\Vert =\frac{\Vert \dot{r}\times \ddot{r}\Vert }{\Vert {\dot{r}}^{3}\Vert }$$

3 $$\tau =\Vert \vec{\tau }\Vert =\Vert \frac{d\vec{B}}{ds}\Vert =\frac{\Vert (\dot{r}\times \ddot{r})\cdot \dddot{r}\Vert }{{\Vert \dot{r}\times \ddot{r}\Vert }^{2}}$$

where, $\vec{T}$ is the tangent vector and $\vec{B}$ is the binormal vector of the Frenet-Serret frame of the curve. Each residue can then be represented by its respective Cα $\kappa$ and $\tau$ values. The curvature values express how much a given point of a curve deviates from a straight line in comparison to the previous point, i. e. $\kappa =0$ only if the point does not change the tangent vector $\vec{T}$ of the curve. Similarly, the torsion expresses how a given point deviates from a plane in comparison to the previous point, i. e. $\tau =0$ only if the point does not change the binormal vector $\vec{B}$ of the curve. The units of $\kappa$ and $\tau$ are per Å^−1^.

Based on the per-segment classification (Fig. 2B), we defined 53 α-helices mispredicted as canonical π-helices (comprising seven or more residues), 127 π-helices correctly predicted as π-helices (only predicted π-helical segments comprising seven or more residues were considered), and 127 α-helices correctly predicted as α-helices (out of >12,000 correctly predicted α-helices 127 were randomly selected to ensure the balance). These helical segments were analyzed using the differential geometry procedure and the resulting curvature as well as torsion values were averaged for each segment and plotted (Fig. 4A).

### Correction of the CB6133, CB5926, CB513, CASP10, and CASP11 datasets

CB6133 and CB513^19^ are standard datasets used in the development of computational tools for protein secondary structure prediction. The CB6133 dataset is composed of training and test subsets, and therefore can be used on its own in the development process. Alternatively, a CB6133 dataset variant, CB6133_filtered, obtained by removing sequences that exhibit >25% sequence identity to sequences in the CB513 dataset, can be used for training and the CB513 dataset for validation. Recently, after the discovery of duplicates in the original CB6133 dataset, an updated version of it (CB5926) as well as an updated version of its filtered dataset (CB5926_filtered) were released.

As these datasets were constructed using older DSSP assignments, which did not annotate π-helices accurately, they show a low abundance of π-helices (Supplementary Table 2). Consequently, as most secondary structure prediction methods train their models using these datasets, they do not predict π-helices. We therefore decided to correct the annotation of π-helices within these datasets. To this end, we downloaded datasets CB5926, CB5926_filtered, CB6133, CB6133_filtered, and CB513 from https://www.princeton.edu/~jzthree/datasets/ICML2014/ and extracted sequences for all entries. Since the sequences did not contain the corresponding PDB identifiers, we built a sequence database corresponding to crystallographic (resolution less than 2.5 Å) and NMR structures (preference was given to X-ray structures if multiple entries were matched). Subsequently, each sequence from the five datasets was used to search our custom PDB database with BLAST and ideal matches, spanning whole query sequence range, were sorted according to their resolution. In each case, the match with the lowest resolution was used to generate updated secondary structure labels with the aid of the procedure described in “*Construction of the training and test sets”*.

To investigate the effects of these corrections, we re-trained two state-of-the-art secondary structure prediction methods, CNNH_PSS^17^ and DCRNN^16^. Both were trained and tested using the original CB5926_filtered and CB513 datasets, respectively, and their updated variants with corrected secondary structure labels. For testing, in addition to the CB513 dataset, we also used the test set developed for the benchmarking of PiPred as well as CASP10 and CASP11 datasets. Testing for the significance of the difference between the performance of PiPred and the other methods was performed as in^40^ using paired t-test; F1 scores were calculated for the individual sequences containing π-helices.

## Supplementary information

Supplementary Dataset 1

## Data Availability

PiPred is available as a web service (https://toolkit.tuebingen.mpg.de/#/tools/quick2d) and as a standalone software (https://github.com/labstructbioinf/PiPred). Results of the Pfam scan and corrected CB6133, CB5926, CB513, CASP10, and CASP11 datasets can be downloaded from https://lbs.cent.uw.edu.pl/pipred.
