# Supplementary material for: PiPred – a deep-learning method for prediction of π-helices in protein sequences
Source: Sci Rep. 2019 May 3;9:6888. doi: 10.1038/s41598-019-43189-4 (PMC6499831; doi:10.1038/s41598-019-43189-4)
Supplement: Supplementary file 1 — Supplementary Dataset 1 [file 41598_2019_43189_MOESM1_ESM.pdf]

PiPred – a deep-learning method for the prediction of π-helices in protein sequences

Jan Ludwiczak[a,b], Aleksander Winski[a], Antonio Marinho da Silva Neto[a], Krzysztof Szczepaniak[a], Vikram Alva[c], and Stanislaw Dunin-Horkawicz[a,*]

[a] Laboratory of Structural Bioinformatics, Centre of New Technologies, University of Warsaw, Banacha 2c, 02-097 Warsaw, Poland

[b] Laboratory of Bioinformatics, Nencki Institute of Experimental Biology, Pasteura 3, 02-093 Warsaw, Poland.

[c] Department of Protein Evolution, Max-Planck-Institute for Developmental Biology, Max-Planck-Ring 5, 72076 Tübingen, Germany

Supplementary Table 1. Statistics of the data sets created in this study.

**Category**	**Training set** [(a)]	**Test set "7"** [(b, c)]	**Test set "6"** [(b, d)]
Number of sequences	20,295	2,215	449
Number of sequences with canonical π-helices	2,694	291	0
Number of π-helical residues	22,774	2,446	0
Number of π-helical segments	3,032	334	0
Number of sequences with π/α-bulges	0	0	449
Number of π/α-bulge residues	0	0	2,856
Number of π/α-bulge segments	0	0	476

[(a)] Maximal pairwise sequence identity in the training set is 50%. The train set contains only canonical π-helices (seven or more residues and at least two $i \rightarrow i+5$ π-type hydrogen bonds); [(b)] Maximal pairwise sequence identity in the test sets is 30% and none of the test set sequences shows more than 30% sequence identity to the training set sequences; [(c)] A test set used to assess the performance of PiPred in detecting canonical π-helices; [(d)] A test set used to assess the performance of PiPred in detecting π/α-bulges (six-residue-long and containing one $i \rightarrow i+5$ π-type hydrogen bond).

Supplementary Table 2. Statistics of the original datasets and the datasets updated in this work.

	Original datasets				Updated datasets			
**Dataset**	**π count** [a]	**π fraction** [b]	**π seg.** [c]	**π str.** [d]	**π count** [a]	**π fraction** [b]	**π seg.** [c]	**π str.** [d]
CB5926_F [e]	212	0.000184	39	38	5164	0.004473	685	582
CB5926	227	0.000182	42	41	5512	0.004426	731	624
CB513	30	0.000354	6	6	336	0.003964	46	43
CB6133_F [e]	212	0.000179	39	38	5278	0.004460	699	593
CB6133	232	0.000181	43	42	5636	0.004409	746	636
CASP10	5	0.000227	1	1	142	0.006443	18	16
CASP11	0	0.000000	0	0	103	0.005364	14	10

[a] Number of π-helical residues; [b] Fraction of π-helical residues; [c] Number of π-helical segments; [d] Number of structures containing at least one π-helix; [e] "F" denotes filtered datasets (see Methods section of the article for details).

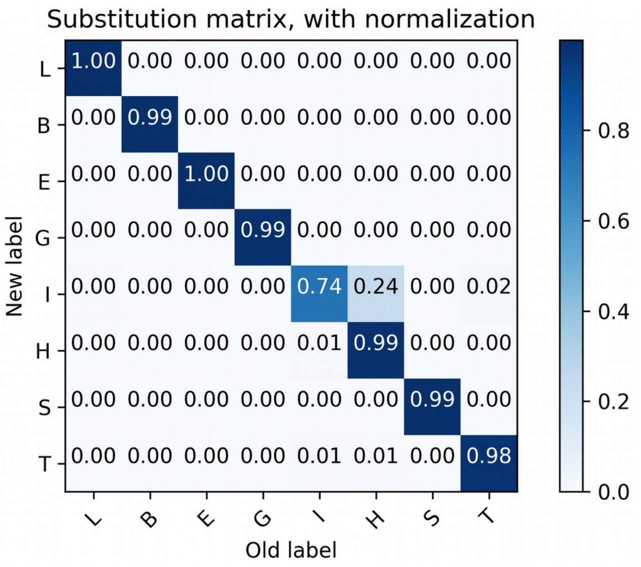

Supplementary Figure 1. Comparison of original and updated CB6133 dataset.
